# Rapid Detection of *Clostridium botulinum* in Food Using Loop-Mediated Isothermal Amplification (LAMP)

**DOI:** 10.3390/ijerph18094401

**Published:** 2021-04-21

**Authors:** Yufei Chen, Hao Li, Liu Yang, Lei Wang, Ruyi Sun, Julia E. S. Shearer, Fengjie Sun

**Affiliations:** 1 School of Grain Science and Technology, Jilin Business and Technology College, Changchun 130507, China; yangliu@jlbtc.edu.cn (L.Y.); wanglei@jlbtc.edu.cn (L.W.); 2 College of Food Engineering, Jilin Engineering Normal University, Changchun 130052, China; lihao@jlenu.edu.cn; 3 College of Life Sciences, Jilin Agricultural University, Changchun 130118, China; 20200926@mails.jlau.edu.cn; 4 School of Science and Technology, Georgia Gwinnett College, Lawrenceville, GA 30043, USA; jshearer@ggc.edu

**Keywords:** *Clostridium botulinum*, botulinum neurotoxin, LAMP, *ntnh*, turbidity method, fluorescence method

## Abstract

Botulinum neurotoxins are considered as one of the most potent toxins and are produced by *Clostridium botulinum*. It is crucial to have a rapid and sensitive method to detect the bacterium *Clostridium botulinum* in food. In this study, a rapid detection assay of *C. botulinum* in food using loop-mediated isothermal amplification (LAMP) technology was developed. The optimal primers were identified among three sets of primers designed specifically based on the partial *ntnh* gene encoding nontoxic-nonhaemagglutinin (NTNH) for rapid detection of the target DNA in plasmids. The optimal temperature and reaction time of the LAMP assay were determined to be 64 °C and 60 min, respectively. The chemical kit could be assembled based on these optimized reaction conditions for quick, initial high-throughput screening of *C. botulinum* in food samples. The established LAMP assay showed high specificity and sensitivity in detecting the target DNA with a limit of 0.0001 pg/ul (i.e., ten times more sensitive than that of the PCR method) and an accuracy rate of 100%. This study demonstrated a potentially rapid, cost-effective, and easy-operating method to detect *C. botulinum* in food and clinical samples based on LAMP technology.

## 1. Introduction

Botulinum poisoning, caused by a botulinum neurotoxin produced by *Clostridium botulinum* (a gram-positive anaerobic spore-forming bacterium), is a peripheral paralysis disease with a high mortality rate [1,2,3]. Botulism is generally categorized into four naturally occurring forms: foodborne botulism, infant botulism, wound botulism, and adult intestinal colonization [4]. Botulinum toxin (BoNT) is not produced by the metabolism of the bacterium itself. The non-toxic precursor toxin (a complex of neurotoxin and either a hemagglutinin or a non-hemagglutinating active protein) is first produced inside the bacterial cells and then released from the autolyzed bacteria into the intestinal tract, where the precursor toxin gains its toxic effects by interacting with trypsin or protein kinases produced by the bacteria [3]. The BoNTs cannot be degraded by gastric acid or digestive enzymes [5], while the toxins are absorbed through the intestine and then enter the blood, acting directly on the nucleus of the brain nerves by preventing the release of acetylcholine, hindering the transmission of nerve impulses and causing muscle paralysis [1].

BoNTs are generally categorized into seven types (A to G), possibly more [6], according to the serological characteristics of its toxin production [7], with more than 40 subtypes [8]. These BoNTs are classified as Category A agents by the Centers for Disease Control and Prevention (CDC) in the United States due to being the most potent of all biological toxins. They are proteins of ~150 kDa, generally existing as one of the structural components of progenitor toxic complexes, either as the M complex (~300 kDa) consisting of BoNT associated with a nontoxic-nonhemagglutinin (NTNH) protein (~150 kDa) or as the L and LL complexes (~500 and 900 kDa, respectively) in which the M complex associates with hemagglutinin proteins [9]. Human botulism is mainly caused by types A, B, and E. BoNTs show some specific geographical distribution in different countries and regions. For example, China has the highest frequency of type A, while type B is predominant in Europe and the eastern United States [10,11]. These different types of toxins express their toxic effects with different mechanisms. For example, Chellapandi and Prisilla studied the interaction between botulinum type A toxin and the intestinal tract to reveal the common toxin transcription and metabolic regulation mechanisms shared among the type A strains [12]. Specifically, the expression of BoNT in type A strains is controlled by the nutritional factors in the intestines. Studies also showed that the neurotoxin is particularly dangerous for children under one year of age, because the normal intestinal flora of infants is not strong enough to reject the botulinum completely, rendering the bacteria the opportunity to grow and reproduce, secrete toxins, and cause poisoning (i.e., infant botulism) [13,14]. The botulinum poisoning in animals is also commonly happening [15,16], including the outbreak of the type C toxin in birds in Incheon, South Korea [17], the identification of the botulinum spores in the honey produced in Republic of Serbia [18] and Poland [19], and the contamination of honey in the Republic of Kazakhstan [20].

Recent studies focused on the structure and the acting mechanisms of the BoNTs. For example, studies showed that the type E toxin is inactivated in low acid foods and phosphate-buffered saline [21]. Moreover, molecular simulation studies on the pathogenesis of the *Clostridium botulinum* strain ATCC3502 revealed the high degree similarity of its proteomes with humans [22], while a mathematical model was used to demonstrate that strain ATCC3502 was used to recognize the potential drug targets due to the lack of functional homologs of the neurotoxin in the host [23]. Studies also demonstrated the medical applications of these neurotoxins. For example, studies of the high-resolution crystal structure of binding domains A3 and A4 in the neurotoxin of *C. botulinum* revealed their subtle differences from other binding domains, providing opportunities of making novel specific therapeutic neurotoxins [24]. Furthermore, several types of biosensors were designed and commercialized based on the optical and electrochemical characteristics of detecting the BoNTs [25,26].

The most common form of botulism is foodborne, generally caused by consumption of contaminated food due to poor processing or temperature control of processed foods [27,28,29]. It is reported by the CDC that ~15% of the 145 botulism cases reported in 2011 were foodborne (CDC Report 2011). Due to the highly toxic nature and frequent occurrence of these neurotoxins, it is critical to screen and detect *C. botulinum* rapidly in order to prevent further outbreak of the pathogens [30,31,32,33,34,35,36]. Moreover, it is constantly demonstrated that it is imperative to have the kits available for quick, initial high-throughput screening of the presence of the pathogens and for further control of the potential pandemic of any infectious diseases, such as the well-known SARS outbreak in 2003 and the current COVID-19 outbreak. Therefore, major botulism outbreaks could be prevented by implementing prevention-based food safety controls, including the identification of *C. botulinum* in a timely manner. Traditionally, the enzyme-linked immunosorbent assay (ELISA) was used to detect *C. botulinum* neurotoxins in various samples [37,38]. To date, PCR has shown several advantages among the various methods used to detect the BoNT-producing bacteria [31,33,39,40,41,42,43]. For example, real-time fluorescent quantitative PCR technology was used to quickly identify the bacteria by detecting the gene *bont* coding for the neurotoxins with high sensitivity [30,39,44,45,46,47,48,49,50,51,52]. The PCR detection methods are generally favored due to their high speed, high sensitivity, high-throughput, and direct amplification and detection of DNA without the limitation by the bacterial survival status in the samples [33], while the disadvantages of PCR methods include the complicated procedures and requirements of professional operation, leading to their limited applications. Furthermore, the DNA extraction methods were optimized for the detection of *C. botulinum* in environmental samples [53].

The loop-mediated isothermal amplification (LAMP) technology is capable of amplifying DNA fragments under isothermal conditions [54]. Since its invention, the LAMP technology was significantly improved from many aspects and was established as the rapid, simple, and cost-effective alternative method to complement PCR techniques [55,56,57,58]. The LAMP reaction needs up to four or six primers (a pair of external primers, a pair of internal primers, and a pair of loop primers), designed specifically based on the conserved regions of the target sequence to identify specifically six or eight independent regions on the target sequence, and initiates a self-circulating chain displacement reaction with the Bst large fragment polymerase. A large amount of target DNA is synthesized in ~60 min under 60 to 65 °C with a by-product of white magnesium pyrophosphate precipitated [54,59]. Due to its high specificity, constant working temperature, high speed, and high sensitivity [60,61,62], the LAMP technology has been widely used (as reviewed in [63]) in detections of bacteria and viruses [64,65,66], drug resistance genes [57,58], parasites [67], and fetal gender identification [68]. Furthermore, the LAMP technology also showed great potential in rapidly detecting pathogenic bacteria in food. For example, the detection of artificial contamination of *Bacillus cereus* in cow milk was completed in ~20 min by using the real-time fluorescent LAMP [69].

The purpose of our study was to establish a novel laboratory method to detect *C. botulinum* (i.e., the *bont* gene clusters) rapidly in various types of food samples using the LAMP technology. Specifically, we designed and identified the optimal primers for the LAMP reactions to amplify a partial DNA fragment (557 bp) of the NTNH coding gene *ntnh* of the *C. botulinum* located immediately upstream of the gene *bont*, which encodes BoNT as the target sequence, in order to detect the bacteria (i.e., via the presence of the *bont* gene cluster), instead of using the bacterium itself (due to the lack of access to the most potent toxin-producing bacteria and the safety concern of culturing these bacteria in the laboratory). We further optimized the LAMP assay to determine the optimal temperature and reaction time of the LAMP reactions, determined the specificity and sensitivity of the primers designed for the LAMP assay, and verified the established LAMP method in various types of food samples. To our knowledge, this is the first report on the detection of *C. botulinum* based on the LAMP technology using gene *ntnh* as the DNA marker. We expected that the optimized working conditions of this LAMP assay could be implemented for further construction of kits for quick, initial high-throughput screening of food and clinical samples.

## 2. Materials and Methods

### 2.1. Reagents and Instruments

Reagents (of analytical purity) Chelex-100 and betaine were purchased from Sigma, USA; manganese chloride, magnesium sulfate, potassium chloride, sodium hydroxide, EDTA, and ammonium sulfate from the Sinopharm Chemical Reagent Co., Ltd. (Shanghai, China); Tris-HCl from the Shanghai Shengke Biotechnology Co., Ltd. (Shanghai, China); Triton X-100 from the Beijing Meilaibo (MyLab) Medical Technology Co., Ltd. (Beijing, China); dNTPs from Pharmacia, USA; NP-40 from Fluka, USA; 2× Tap MIX Kit from the Tiangen Biochemical Technology Co., Ltd. (Beijing, China); Agarose from Amresco, USA; LAMP DNA Amplification Kit, LAMP reaction tube, and calcein (the fluorescent detection reagent) from the Eiken Chemical Co., Ltd. (Tochigi, Japan); the real-time turbidity meter (LA-320C) from the Rongyan Chemical Co., Ltd. (Tokyo, Japan); thermostatic metal bath (HB-2) from the Wealtech Corporation (Sparks, NV, USA); Spectrophotometer (NanoQ^TM^) from the CapitalBio Technology Co., Ltd. (Beijing, China); gel imaging system (Quantun -ST5) from the Gel imager VILBER (Beijing, China); and PCR thermal cycler (ETC811) from the Gene Amplifier Suzhou Dongsheng Xingye Scientific Instrument Co., Ltd. (Suzhou, China).

### 2.2. Selection of Target DNA Fragment

The NTNH coding gene *ntnh* of *Clostridium botulinum* was chosen as the target DNA marker for examination of the specificity tests [47,70,71]. Both the genes encoding BoNT and NTNH were previously used as the target DNA markers in detection of the bacterium *C. botulinum*, as indicated by the presence of the *bont* gene clusters [15,20,33,39,48,49,50,51,56,71,72]. Studies showed that the gene *ntnh* is a universally present component of *bont* gene clusters and is present in all bacterial strains producing BoNTs but absent from non-toxic strains [73,74]. Due to its highly toxic nature, the access to *C. botulinum* is extremely limited in China. A partial DNA fragment (557 bp) of gene *ntnh* identified by the bioinformatic analysis at the National Center for Biotechnology Information (https://www.ncbi.nlm.nih.gov/ (accessed on 15 April 2021)) was artificially synthesized and purified by using the phosphoramidite method (Sangon Biotech, Shanghai, China). The nucleic acid sequence was subcloned to the linearized vector pMD18-T and then transformed into *E. coli* DH5α. The target DNA fragment was ligated into the plasmid pMD18-T (50 ng/μL) using the following system: T4 DNA Ligase 1 μL (Sangon Biotech, Shanghai, China), 10× ligation buffer 2 μL, DNA 2.14 μL (50 ng/μL), pMD18-T Vector 1 μL (50 ng/μL), H_2_O 13.86 μL, in a total volume of 20 μL, cultured under 16 °C for 4 h. The plasmids were then transformed into *E. coli* DH5α for storage. To confirm the successful transformation of the plasmids into *E. coli* DH5α, a pair of primers was designed to amplify the target sequence using PCR (forward primer 5′-GGCGAATCTTTGAGTATAGATG-3′ and reverse primer 5′-TTTCTACTATGTTTGCTCCTGG-3′) with the PrimeSTAR HS DNA Polymerase (Takara, Dalian, China). The PCR amplification procedure was as follows: The DNA template was pre-denatured for 5 min and denatured for 30 s at 94 °C, annealed for 50 s at 55 °C, extended for 40 s at 72 °C, and finally extended for 10 min at 72 °C after 35 cycles. The PCR products were examined using 1% agarose gel electrophoresis and sequenced using either the above forward or reverse primers (Sangon Biotech, Shanghai, China).

### 2.3. Design and Determination of the Optimal LAMP Primers

The LAMP primers based on the target DNA fragment of gene *ntnh* described above were designed using the LAMP primer design software Primer Explorer (http://primerexplorer.jp/e/, accessed on 12 July 2017). Three groups of LAMP reactions with three sets of primers (RD1, RD2, and RD3) were designed based on the six independent regions of the botulinum-specific target DNA sequence (Table 1). Two loop primers were designed to accelerate the amplification reactions (Shanghai Biotech Biotechnology Co., Ltd. Shanghai, China).

### 2.4. LAMP Reactions

The 25-μL LAMP reaction mixtures contained 20 mM Tris-HCl (pH 8.8), 10 mM KCl, 10 mM (NH_4_)_2_SO_4_, 0.1% Triton X-100, 0.8 M Betaine, 8 mM MgSO_4_, 1.4 mM dNTP, 8U *Bst* DNA polymerase, 40 pmol primers FIP and BIP, 5 pmol primers F3 and B3, 20 pmol primers LB and LF, and 1 μL template DNA with varied concentrations based on different samples. The mixtures were set in the LAMP reaction tubes at a constant amplification temperature of 60–65 °C for 60 min. The optimal primers were selected based on the time of amplification of LAMP reaction and the concentration of the amplified DNA templates. Each reaction was performed in triplicates.

### 2.5. Detection of the Amplification Products of LAMP Reactions

The LAMP products were detected using two methods. First, the direct visual inspection was based on the change of the fluorescence of the LAMP reaction containing 1 µL of calcein [75,76]. A positive reaction was indicated by the color change from orange to green in the reaction, while a negative reaction was indicated by the failure of color change from orange to green. The advantage of the fluorescence detection was that the color change of the LAMP reactions was observed directly either by the naked eye under natural light or the UV light. Second, in the detection of turbidity method [22], the amplification products of the LAMP assay were examined using spectrophotometry. Specifically, the value of the optical density at 650 nm (OD_650_) of the magnesium pyrophosphate (Mg_2_P_2_O_7_) produced in the LAMP reactions was measured every 6 s using a Loopamp real-time turbidimeter (LA-320C, Eiken Chemical Co., Ltd., Tokyo, Japan) and was plotted against reaction time to generate the curve to determine the positive or negative reactions.

### 2.6. Determination of the Optimal Temperature for LAMP Reaction

The established LAMP reaction system using the optimal LAMP primers was used to identify the optimal temperature for the LAMP reactions. The temperature of the LAMP reactions was set to 60, 61, 62, 63, 64, 65, 66, and 67 °C, respectively, with the LAMP reaction time set to 60 min. The OD_650_ values of the reactions were measured every 6 s as described above.

### 2.7. Specificity of LAMP Reactions

The specificity tests were carried out based on the above established LAMP reaction system with the optimal primers and reaction temperature of 64 °C using a total of 30 species of bacteria, with 14 being pathogenic, 9 being non-toxic species in the genus of *Clostridium*, and 7 being taxonomically closely related to *Clostridium* (Table 2; CapitalBio Technology Co., Ltd., Beijing, China). The target DNA fragment ligated in the plasmids and the double-distilled water were used as the positive and reaction controls, respectively. These 30 strains of bacteria were provided by the CapitalBio Technology Co., Ltd. (Beijing, China). The culture and the extraction of genomic DNA of these bacteria were completed in the P3 laboratory of the CapitalBio Technology Co., Ltd. (Beijing, China) based on standard protocols. The genomic DNA of the bacteria was extracted using the Chelex method. Specifically, the equal volume of both the bacterial pellet in 200 µL phosphate-buffered saline (PBS) and the Chelex DNA extraction buffer (25 mM NaOH, 10 mM Tris-HCl, 1% Triton X-100, 1% NP-40, 0.1 mM EDTA, and 2% Chelex-100) were mixed and incubated at 100 °C for 10 min and cooled immediately on ice. The supernatant collected from the centrifugation at 14,000× *g* for 2 min was used as the template in further amplifications of the LAMP assay and PCR method. The genomic DNA was also prepared by 10-fold serial dilutions to give concentrations ranging from 1 ng/µL to 0.000001 pg/µL as needed.

### 2.8. Sensitivity of LAMP Assay and PCR Method

The sensitivity of the above established LAMP assay was examined using target DNA fragment with 10-fold serial dilutions after quantification. Double-distilled water was used as a reaction control. The amplified products of LAMP reaction were detected using both the fluorescence and the turbidity methods, as described above. The mixtures of the 25-µL volume PCR contained 12.5 µL Taq mix reagents, 1 pmol primers F3 and B3, and 1 µL DNA template with 10-fold serial dilutions. The PCR amplification cycles included: pre-denaturation at 95 °C for 5 min, followed by 30 cycles of denaturation at 95 °C for 30 s, annealing at 55 °C for 30 s, and extension at 72 °C for 30 s, and a final extension at 72 °C for 7 min. Agarose (1%) gel electrophoresis under 120 V for 35 min was used to examine the PCR-amplified products, which were then stained with ethidium bromide and imaged to identify the sensitivity of PCR method in the detection of *Clostridium botulinum.*

### 2.9. Validation of the Optimized LAMP Assay and Potential Kit Assembly

The established LAMP assay was evaluated using a total of 24 samples, including eight samples of Korean kimchi (i.e., preserved cabbage) and two samples of preserved meat (pork), bacon, sour meat (pork), salami (pork), ham (pork), fish product (canned sardine), soybean paste, and dairy product (powdered cow milk), respectively, purchased from the local grocery stores with the target DNA fragment as the positive control. Genomic DNA was extracted from these samples using various types of DNA extraction kits (e.g., Qiagen, Takara, Sigma-Aldrich, and Fisher Scientific) according to manufacturer’s instructions (Table 3). The amplification products of LAMP reactions were detected using the turbidity method, as described above. The overall performance of this LAMP assay was assessed using the national food safety standards in China (GB/T 4789.12-2016). The samples showing the positive LAMP reactions were further confirmed by 16S rRNA sequencing, and the 16S rRNA DNA sequences were used to carry out Basic Local Alignment Search Tool (BLAST) analysis at NCBI (https://www.ncbi.nlm.nih.gov/ accessed on 15 April 2021). Specifically, the concentration and purity of the genomic DNA were determined using the spectrophotometer (NanoDrop 2000) and 1% agarose gel electrophoresis. The mixtures of 50-μL volume of PCR contained PrimeSTAR HS DNA Polymerase 25 μL, 16S rRNA universal primer-27F (5′-AGAGTTTCCTTGGCTCAG-3′) 1 μL and primer-1492R (5′-ACGGHTACCTTGTTTGGACTT-3′) 1 μL, genomic DNA 1 μL, and double-distilled H_2_O 22 μL. The PCR amplification procedure was as follows: 94 °C pre-denaturation for 5 min, followed by 35 cycles of 94 °C denaturation for 30 s, 55 °C annealing for 50 s, 72 °C extension for 40 s, and a final extension of 72 °C for 10 min. The PCR product (5 μL) was examined using 1% agarose gel electrophoresis. The purified PCR products were sequenced using either primer-27F or primer-1492R by the CapitalBio Technology Co., Ltd. (Beijing, China). The construction of the chemical kits for the rapid detection of *Clostridium botulinum* in the food or clinical samples was recommended based on the optimized conditions of the LAMP reaction established in our study.

## 3. Results

### 3.1. The Optimal Primers for LAMP Reactions

**Figure 1:**
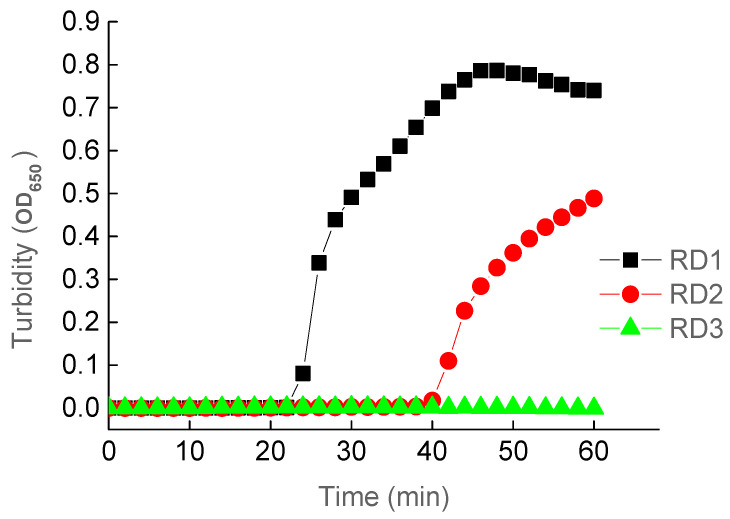
LAMP Reactions Using Three Sets of Primers (RD1, RD2, and RD3).

The results of the LAMP assays using primer sets RD1 and RD2 showed positive reactions, while RD3 failed to amplify the target DNA fragment (Figure 1). Primer set RD1 was identified as the most optimal because this set of primers was faster (~20 min) than RD2 to amplify the target DNA. The higher efficiency of the LAMP reactions using RD1 was indicated by the higher OD_650_ values (~45 min) than that of RD2 measuring the magnesium pyrophosphate (Mg_2_P_2_O_7_) generated in the amplifications of the target DNA fragment. Therefore, the optimal primer set RD1 was used in the subsequent LAMP reactions. These results demonstrated that the use of loop primers in RD1 significantly shortened the amplification time by ~50%. Previous studies showed similar results—that the application of loop primers decreased the LAMP reaction time [77]. These studies further demonstrated the importance of using the loop primers in the LAMP reactions.

### 3.2. The Optimal Temperature for LAMP Reactions

**Figure 2:**
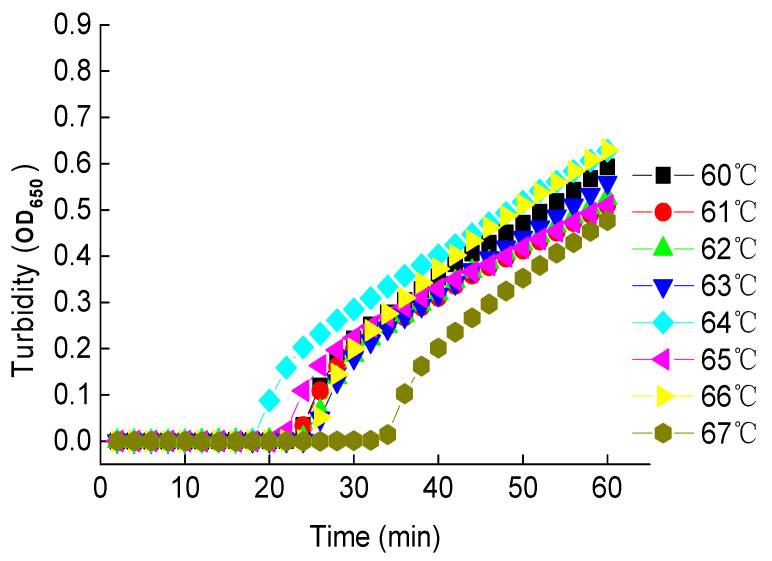
The LAMP Reactions Under Different Temperatures.

Using the optimal primer set RD1, LAMP reactions under various temperatures were carried out to determine the optimal temperature for LAMP assays. Results showed that the fastest amplification (~18 min) was obtained under 64 °C with a reaction time of 60 min, while the slowest amplification (~35 min) was observed under 67 °C (Figure 2). The amplification times fell between 20 and 24 min under other temperatures investigated. The highest OD_650_ values were observed in 60 min under all of these temperatures. Therefore, the optimal temperature and reaction time were set to 64 °C and 60 min, respectively, for further LAMP reactions. This temperature fell into the general range of optimal temperatures from 60 to 70 °C, while this reaction time was generally used in LAMP reactions. It was noted that plots showing the OD_650_ values vs. reaction time did not reach the plateau in 60 min. It was expected the amplification reactions would continue given the extended reaction time and the sufficient amount of ingredients loaded in the LAMP reaction tube. However, in 60 min, the concentration of magnesium pyrophosphate precipitated was sufficient enough to indicate the positive amplification of the LAMP reaction; therefore, it was not necessary to extend the reaction time beyond 60 min.

### 3.3. Specificity of the LAMP Assay

**Figure 3:**
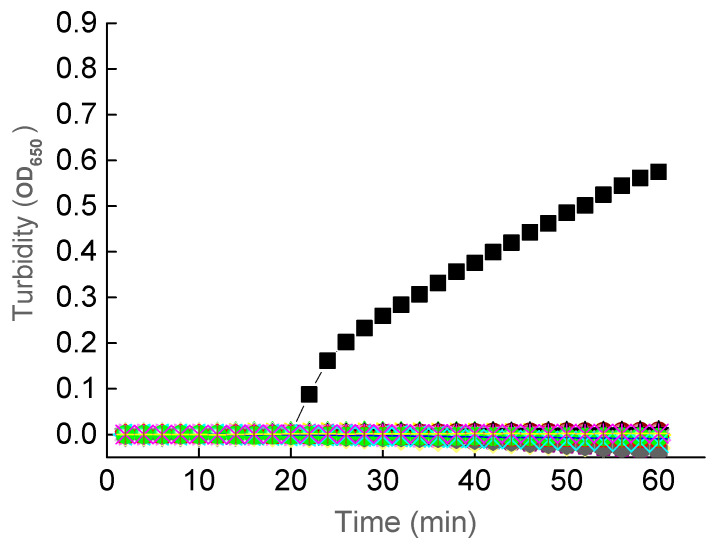
Specificity of the LAMP Assay Examined Using the Turbidity Method. Symbols of Black Squares Indicate the Positive Control Containing the Target DNA Fragment. No Amplification Is Observed in the 30 Strains of Bacteria (Table 2) and the Reaction Control

**Figure 4:**
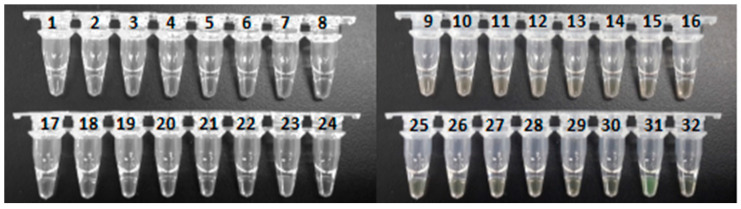
Specificity of the LAMP Assay Examined Using the Visible Fluorescence Method. The Numbers of the Samples Are the Same as Those Listed in Table 2. Samples 31 and 32 Are Positive and Reaction Controls, Respectively.

The specificity of the LAMP assay was examined by using the plasmid containing the target DNA fragment as the positive control and the double-distilled water as the reaction control, with the LAMP reaction time set to 60 min and reaction temperature of 64 °C. As expected, results showed that the 30 non-botulinum strains of bacteria and the reaction control did not show the amplification of the target DNA fragment, which was amplified in the positive control in 20 min using primer set RD1 (Figure 3). The results of the specificity experiments were also examined using the fluorescence method (Figure 4). Again, it was clearly observed that only the positive control changed color from orange to green. These results of both turbidity method and fluorescence visual inspection demonstrated the high specificity of the established LAMP assay under the optimized reaction conditions.

Previous studies using PCR technology extensively utilized the *ntnh* gene as the DNA marker to detect the *bont* gene clusters with high specificity and sensitivity [15,19,20,48,50,51,70]. Studies using LAMP technology to detect the BoNT-producing *Clostridium botulinum* types A and B showed high specificity with the *bont* genes [56]. In comparison to the study by Sakuma et al. [56], we used the partial DNA fragment of gene *ntnh* as the DNA marker to detect the *bont* gene clusters because the *ntnh* gene is located immediately upstream of the gene *bont*. These results indicated that both the BoNT and NTNH genes are appropriate DNA markers to be used in either the PCR technology or the LAMP reaction to detect *Clostridium botulinum.* It was noted that because the target DNA fragment was artificially synthesized based on the conservative region of the *ntnh* gene, the specificity of these results was further confirmed by the 16S rRNA sequencing in the verification below.

### 3.4. Sensitivity of the LAMP Assay and PCR

**Figure 5:**
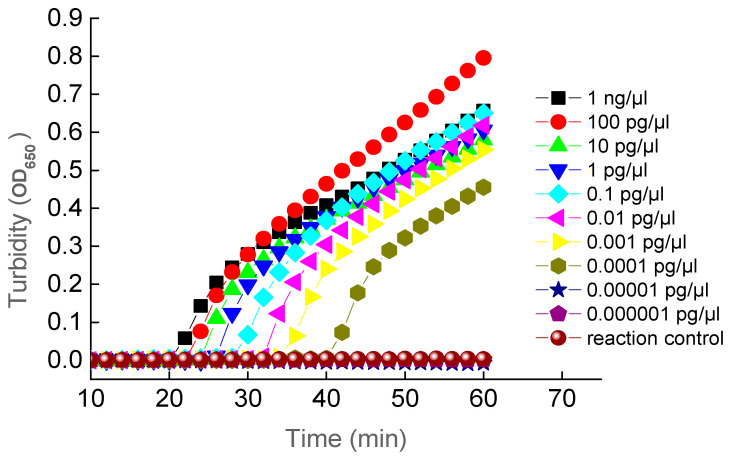
Sensitivity of LAMP Assay under Various Concentrations of the Target DNA Examined Using Turbidity Method.

**Figure 6:**
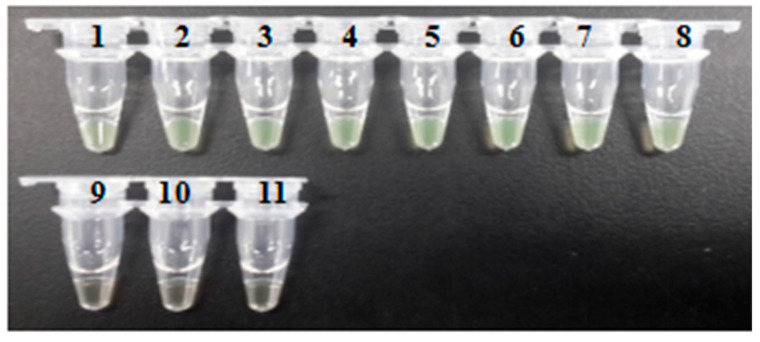
Sensitivity of the LAMP Assay Examined Using Visible Fluorescence Method. The Concentrations of the Target DNA in Samples 1, 2, 3, 4, 5, 6, 7, 8, 9, and 10 are 1 ng/µL, 100 pg/µL, 10 pg/µL, 1 pg/µL, 0.1 pg/µL, 0.01 pg/µL, 0.001 pg/µL, 0.0001 pg/µL, 0.00001 pg/µL, and 0.000001 pg/µL, Respectively. Sample 11 is the Reaction Control.

**Figure 7:**
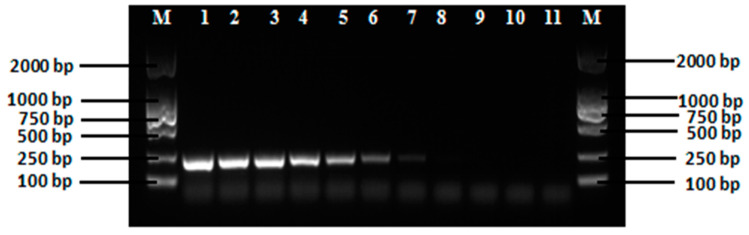
Sensitivity of PCR using Primers F3 and B3 with the Target DNA Fragment of 222 bp. The Concentrations of the Target DNA in Lanes 1, 2, 3, 4, 5, 6, 7, 8, 9, and 10 are 1 ng/µL, 100 pg/µL, 10 pg/µL, 1 pg/µL, 0.1 pg/µL, 0.01 pg/µL, 0.001 pg/µL, 0.0001 pg/µL, 0.00001 pg/µL, and 0.000001 pg/µL, Respectively. Sample 11 is the Reaction Control. Lane M Contains DNA Markers.

The sensitivity of the established LAMP reactions was examined by diluting the target DNA to a series of concentrations ranging from 1 ng/µL to 0.000001 pg/µL. These results were examined using both the turbidity method (Figure 5) and the visual fluorescence method (Figure 6) showing that the lowest concentration of the target DNA being amplified by LAMP reactions was 0.0001 pg/µL. The sensitivity of PCR in detecting the target DNA fragment was carried out using primers F3 and B3 with an expected length of amplified DNA fragment of 222 bp. Results showed that the lowest concentration of the target DNA being amplified was 0.001 pg/µL (Figure 7). These data demonstrated that the PCR method showed sensitivity ten times lower than that of the LAMP assay. These results demonstrated that the LAMP assay showed greater sensitivity than that of the PCR method. Previous studies reported similar results with comparable sensitivity as our research. The LAMP technology has generally shown increased sensitivity in comparison to the PCR method [54,56,57,76,78,79].

**Figure 8:**
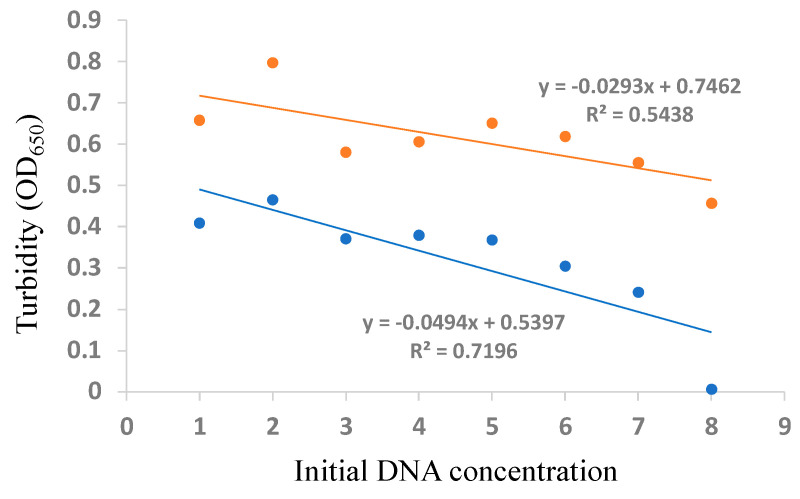
Calibration Curves of *Clostridium botulinum* Detection Based on the Initial DNA Concentration vs. Turbidity in the LAMP Reaction at 60 min (orange dots and line) and 40 min (blue dots and line). The Initial DNA Concentrations of 1,000; 100; 10; 1; 0.1; 0.01; 0.001; and 0.0001 pg/µL are Normalized Based on “log(DNA concentration) + 5” Corresponding to Labels of 1–9, Respectively, on the *x*-axis.

The calibration curves of *C. botulinum* detection based on the initial DNA concentration vs. the turbidity in the LAMP reaction at 40 and 60 min were constructed (Figure 8).

These studies demonstrated clearly that the LAMP assay contained more advantages over the PCR method (Table 4). First, the LAMP assay is operated in an isothermal condition, while the PCR is carried out in a temperature-cycling environment, which is always time-consuming and requires high precision of the PCR instruments. Moreover, the LAMP method is evaluated using the turbidity method in a positive reaction by real-time examination of the reaction mixture with direct visual fluorescence detection without performing the agarose gel electrophoresis. In comparison to the previous studies taking 24 h for the real-time PCR assay to screen enrichment cultures of bacterial strains using *ntnh* gene as the target DNA markers [48], our LAMP assay was completed with a positive detection in less than 90 min, including the extraction of DNA samples (below). Second, the specificity and sensitivity are greatly increased by the LAMP method. This is because the LAMP assay uses the specifically designed primers to recognize the target sequences of four or six of the six to eight independent regions in the target DNA sequence, while PCR primers recognize only two independent regions of the target sequence. Third, studies also showed that the purification of DNA samples is generally not required in the LAMP reactions because it is less susceptible to different components in the samples than the PCR method [80]. Although it was noted that the LAMP method may show a high rate of false positive reactions due to its high amplification efficiency of target DNA fragment [81], this type of potential contamination could be avoided by spatially separating the reagent preparation and the LAMP reactions. We note the limitation of our study. Specifically, the current study was conducted using a relatively small sample size. Therefore, for better comparing the sensitivity and specificity of our proposed method to the PCR approach, we suggest additional experiments in the future with larger sample sizes and a wider array of food products for further comparing the external validity of these testing methods.

### 3.5. Verification of the Established LAMP Assay

**Figure 9:**
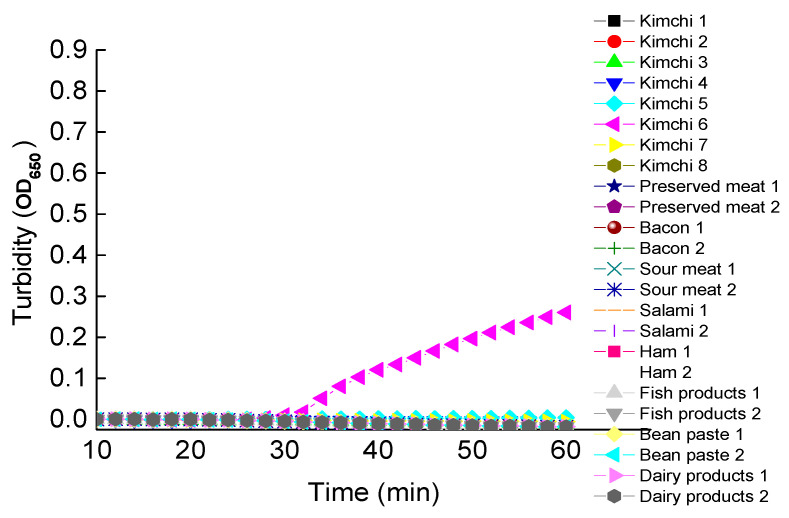
The LAMP Reactions of Eight Samples of Korean Kimchi and Two Samples of Preserved Meat (Pork), Bacon, Sour Meat (Pork), Salami (Pork), Ham (Pork), Fish Product (Canned Sardine), Soybean Paste, and Dairy Product (Powdered Cow Milk), Respectively. The Genomic DNA Concentrations of These Samples are Given in Table 3.

Using the established LAMP assay in this study, a group of 184 food samples purchased from the local grocery stores was tested for the detection of *Clostridium botulinum* (Figure 9; many samples with the absence of *C. botulinum* not shown). Results showed that the amplification (~25 min) was detected in one of the eight samples of Korean kimchi (i.e., sample 6), indicating the possible contamination or presence of *C. botulinum*. The possible presence of *C. botulinum* in this sample of Korean kimchi and the absence in other tested samples are probably due to the different procedures of processing these different types of food. Specifically, Korean kimchi is generally made with substantial exposure to the open air without complete sterilization. Overall, the entire procedure of the LAMP assay was completed with a positive detection in less than 90 min, including the extraction of DNA samples. This sample was further confirmed by 16S rRNA sequencing and the BLAST at NCBI. Results of the nucleotide BLAST search in the NCBI database showed that the 16S rRNA sequence (of 1445 bp in length) derived from the positive Korean kimchi sample 6 was closely related to those of various strains of *C. botulinum* (e.g., NCTC 13319, CFSAN064329, DFPST0029, B515, B609, B305, B742, and F1425) among many of the top hits of the BLAST search with homology over 99%. These results demonstrated the 100% compliance rate based on the food safety standard in China (GB/T 4789.12-2016). It was noted that this LAMP assay could be further verified by using clinical samples. However, due to our limited access to these samples, these verifications could not be completed in this study. It was previously demonstrated constantly that it is crucial to have easy-operating, cost-effective, and reliable kits available for initial high-throughput screening of the pathogens and for further control of the potential pandemic of any infectious diseases, such as the SARS outbreak in 2003 and the current COVID-19 outbreak. Although there are some commercial kits available for the detection of *Clostridium botulinum* (e.g., https://www.coleparmer.com/; https://www.bc-diagnostics.com/; and https://www.kitpcr.com/ accessed on 15 December 2020) [82], limitations of these commercial kits include either limited number of types of toxins being detected with low sensitivity or the involvement of PCR-based operations. Therefore, our development of the novel LAMP assay for quick detection of *Clostridium botulinum* is practically important.

## 4. Conclusions

Development of a fast and reliable protocol to detect pathogens is always crucial for early diagnosis and control of a potential outbreak. We established a novel LAMP assay to detect the BoNT-producing bacterium *Clostridium botulinum* quickly. Furthermore, this method is easy to operate and cost-effective, showing high specificity and sensitivity in comparison to the PCR method. In our establishment of this novel detecting method, instead of using the bacterium itself (due to lack of access to the most potent toxin-producing bacteria), we identified a partial DNA fragment (557 bp) of the NTNH coding gene *ntnh* of the *Clostridium botulinum* located immediately upstream of the gene coding for BoNT as the target sequence for examination of the specificity and sensitivity of the LAMP assay. The optimal primers, temperature, and reaction time were determined to be RD1, 64 °C, and 60 min, respectively. The validity of the method was confirmed by using this LAMP assay to detect the presence of *Clostridium botulinum* in one of the 16 Korean kimchi samples tested. These optimized conditions for the LAMP assay could be adopted to make chemical kits for quick initial high-throughput screening of *Clostridium botulinum* in food and clinical settings.

## Figures and Tables

**Table 1:** *Clostridium botulinum* Specific Primers Used in LAMP Reactions.

Primer Sets	Primer Names and Types	DNA Sequences (5′ to 3′)
RD1	F3 (outer forward primer)	AAGTATAAATTCCCCGGTAGA
	B3 (outer backward primer)	GCTTGTAAGAACTTATCTTTTTCAC
	FIP (forward inner primer)	TGGGAGCAACCTTAAAAGCCTTAAAAAATGTTGTAGTAGTTAGAGCT
	BIP (backward inner primer)	GGTGGCGCCAGAGAGATATTAGAATCATATATTCCCCCATCAA
	LF (loop forward primer)	AAACCGTATCAGTTTTTCT
	LB (loop backward primer)	ATGGCGAATCTTTGAGTATA
RD2	F3 (outer forward primer)	GTTCTTACAAGCCATTATTACTTTG
	B3 (outer backward primer)	AAGGAAATGGAATAGTACTTGAA
	FIP (forward inner primer)	ATCCATAAGGAAATGGAATAGCTGTAAAGAATTAATAGTACTAACGCTGG
	BIP (backward inner primer)	TATAGGTGGAGGGTATTATGCACCTTTATTAGATTTTGGTGCTGATCC
RD3	F3 (outer forward primer)	ATGATTCTAATTTTCTTTCACAAGA
	B3 (outer backward primer)	GATTTTGGTGCTGATCCAA
	FIP (forward inner primer)	TCCCCAGCGTTAGTACTATTAATTCTGTGAAAAAGATAAGTTCTTACAAGC
	BIP (backward inner primer)	TGATTTCTACAGCTATTCCATTTCCTTCATATTAGGTGCATAATACCCTC

**Table 2:** Thirty Strains of Bacteria Selected for Specificity Test of LAMP Reactions.

Number of Bacteria	Species of Bacteria
1	*Shigella flexneri* ATCC12022 *
2	*Escherichia coli* ATCC25922 *
3	*Yersinia enterocolitica* ATCC23715 *
4	*Enterococcus faecalis* ATCC29212 *
5	*Pseudomonas aeruginosa* 10211 *
6	*Staphylococcus aureus* 1897/2722 *
7	*Streptococcus dysgalactiae* ATCC12388 *
8	*Bacillus cereus* ATCC11778 *
9	*Streptococcus pneumoniae* 31002 *
10	*Enterococcus faecium* ATCC29212 *
11	*Vibrio Parahemolyticus* ATCC17802 *
12	*Staphylococcus epidermidis* 1.2429 *
13	*Salmonella enterica* ATCC14028 *
14	*Streptococcus uberis* ATCC700407 *
15	*Clostridium tetani* ATCC19406
16	*Clostridium perfringens* NCTC8798
17	*Clostridium difficile* ATCC9689
18	*Clostridium beijerinckii* NCIMB8052
19	*Clostridium acetobutylicum* ATCC824
20	*Clostridium sporogenes* ATCC19404
21	*Clostridium sordellii* ATCC9714
22	*Clostridium butyricum* ATCC25755
23	*Clostridium tertium* CICC10820
24	*Bntorobater sakazakii* ATCC29544
25	*Clostridium bifermentans* CICC22952
26	*Clostridium thermosaccharolyticum* ATCC7956
27	*Bacillus stearothermophilus* ATCC7953
28	*Bacillus thuringiensis* LSZ9408
29	*Bacillus subtilis* ATCC6633
30	*Bacillus megaterium* DSM90

Symbol “*” after the strain names indicates a pathogenic species. All these strains were obtained from the CapitalBio Technology Co., Ltd. (Beijing, China).

**Table 3:** A Total of 184 Food Samples Tested Using the LAMP Assay Established in This Study.

Sample	Genomic DNA Concentration (ng/μL)
Korean kimchi 1–16	1.8–45.2
Preserved meat (pork) 1–8	143.5–154.6
Bacon 1–8	89.5–124.2
Sour meat (pork) 1–8	123.5–180.1
Salami (pork) 1–8	125.2–165.6
Ham (pork) 1–8	68.5–95.6
Fish product (canned sardine) 1–8	105.5–127.3
Soybean paste 1–8	176.2–275.2
Dairy product (powdered cow milk) 1–8	125.8–151.1
Pork rib 1–8	106.2–143.5
Fish product (canned anchovy) 1–8	51.8–70.1
Fish product (canned tuna) 1–8	19.6–26.9
Fish product (canned salmon) 1–8	82.5–95.4
Fish product (canned yellow croaker) 1–8	29.8–40.1
Stinky tofu 1–8	215.1–235.7
Dried tofu 1–8	135.9–158.7
Vegetarian meat 1–8	60.8–73.5
Vegetarian cow stomach 1–8	40.2–51.1
Vegetarian ham 1–8	74.6–92.7
Quail egg (canned) 1–8	28.9–41.7
Chicken foot (canned) 1–8	50.2–67.1
Honey 1–8	95.2–105.2

**Table 4:** Comparison of the Efficiency of LAMP and PCR Methods in Detecting *Clostridium botulinum* in Food Samples.

Condition	LAMP	PCR
Temperature of reaction	Isothermal	Cycling
Time of completion	Short (~90 min)	Long (~24 h)
Detection method	Direct examination of turbidity in a positive reaction with visual fluorescence	Requirement of high precision of detection on the agarose gel electrophoresis
Specificity and sensitivity	Increased by specifically designed primers to recognize four or six independent regions in the target DNA sequence	PCR primers recognize only two independent regions of the target sequence
Purification of DNA sample	Not required due to its less susceptibility	Required

## Data Availability

The data presented in this study are available on request from the corresponding authors.
